# A high-resolution temporal transcriptomic and imaging dataset of porcine wound healing

**DOI:** 10.1038/s41597-025-05921-w

**Published:** 2025-10-10

**Authors:** Ksenia Zlobina, Hsin-ya Yang, Manasa Kesapragada, Fan Lu, Anthony Gallegos, Guillermo Villa-Martinez, Moyasar A. Alhamo, Kan Zhu, Cynthia Recendez, Craig Collins, Marco Rolandi, Athena Soulika, Elham Aslankoohi, Min Zhao, Marcella Gomez, R. Rivkah Isseroff

**Affiliations:** 1 University of California Santa Cruz, Department of Applied Mathematics, Santa Cruz, 95064 USA; 2 University of California Davis, Department of Dermatology, Sacramento, 95817 USA; 3 University of California Davis, Department of Ophthalmology and Vision Science, Sacramento, 95817 USA; 4 University of California Santa Cruz, Department of Electrical Engineering, Santa Cruz, 95064 USA; 5 Shriners Hospitals for Children, Pediatric Regenerative Medicine, Sacramento, 95816 USA; 6 VA Northern California Health Care System, Dermatology Section, Mather, CA 95655 USA

**Keywords:** Physiology, Translational research, Medical imaging

## Abstract

Wound healing is a dynamic process involving various cell types. Collecting samples from healing wounds and investigating their transcriptomics can provide deeper insights into the underlying processes. In recent years, several experiments have been conducted to gather transcriptomic data from wounds in both humans and animals. However, the temporal resolution of such data often does not adequately match the dynamics of the process, and spatial aspects are frequently overlooked. Here, we present a dataset collected from an experiment on wound healing in pigs, including gene expression profiles at the wound edge and center, and photographs of the wounds. Photographs provide non-invasive data, and advancements in image analysis using artificial intelligence methods are actively being integrated into medical practice. Being collected within the same experiment, these comprehensive data can aid in building intelligent wound diagnostics and treatment algorithms.

## Background & Summary

The understanding of the process of wound healing has progressed over time, and with it the classification of phases of healing have evolved. Perhaps first described in the 1970’s^1,2^, a four-phase model of wound healing is now commonly accepted that encompasses overlapping phases of hemostasis, inflammation, proliferation and maturation/remodeling. With advances in cellular and molecular technologies, more details of each phase continue to be revealed. To create the “ground truth” that characterizes and, indeed, defines each phase of the healing curve, it is essential to simultaneously and sequentially monitor multiple cellular and molecular responses in the wound tissue over the course of healing. However, the collection of wound tissue multiple times over the course of healing from human subjects is not feasible. Although this has been examined in rodent models^3–5^, rodent skin has many dissimilarities to human skin making it a less relevant model. Instead, pigs have evolved as the preferred model for wound healing studies.

Porcine skin has similar architecture and healing processes to human skin, which can better predict the potential for human therapeutics and address safety concerns^6^ and is the preferred model for studies of wound healing recommended by the FDA^7^ and widely used by investigators^8–12^. For these reasons, the pig model was selected for this study that fully elucidates the molecular, cellular and morphological events that characterize the phases of healing of an excisional skin wound. Ongoing research into the pig transcriptome highlights its utility as a model for human biology, particularly in understanding tissue-specific gene expression and regulation^13^.

The recent advances in the field of artificial intelligence (AI) give hope that, once more closely integrated into medical practice, they will be able to improve diagnostics and treatment. Utilizing AI or machine learning algorithms to handle large datasets collected from images, or transcriptomic and proteomic analysis could maximize the information that can be extracted and generate predictive models. However, to create the training models for the AI algorithms, vast amounts of data are needed to establish the “ground truth.” Developing AI approaches for wound diagnosis and treatment would not only help to solve issues in delayed wound healing but could also alleviate the labor requirement and the financial and social burdens associated with wound care.

In this work, our goal is to integrate a complete set of wound images and RNAseq samples from wound tissue throughout the entire healing process. The corresponding, temporal information will create a comprehensive characterization of the normal healing of an excisional wound in a clinically-relevant pig model. We anticipate that the wound datasets shared in the current work will facilitate development of predictive algorithms not only for pig wounds, but more importantly for the human patient.

## Methods

### Wounding and sample collection

The wounding was carried out by the DaVinci Biomedical Research Products Inc, contract laboratory (IACUC protocol number DB-749). Six domestic pigs (Yorkshire-mix breed, females, 45–50 Kg) were utilized. A total of 12 full-thickness, circular excisional wounds were created (6 on each side of the dorsum) in each animal, 2 cm in diameter. Two layers of wound dressing, Telfa Clear and Tegaderm, were applied to the wounds. No other treatment was administered. Baseline (Day 0) wound images, and the excised skin tissue was collected.

During the post-operative period, 3 out of 6 animals were anesthetized on post-operative days 1, 2, 3, 4, 5, 6, 7, 9, 11, 13, 15, 16, 19, and 21. Wound images were captured using a DSLR camera. The camera was positioned at a fixed distance of 1-foot above the wounds using a mount for imaging. A Medline NE1 Wound Assessment Tool (photo scale) was used for calibration in each image. Wound images were captured on intact wounds only (those not previously subjected to tissue sampling).

**Fig. 1:**
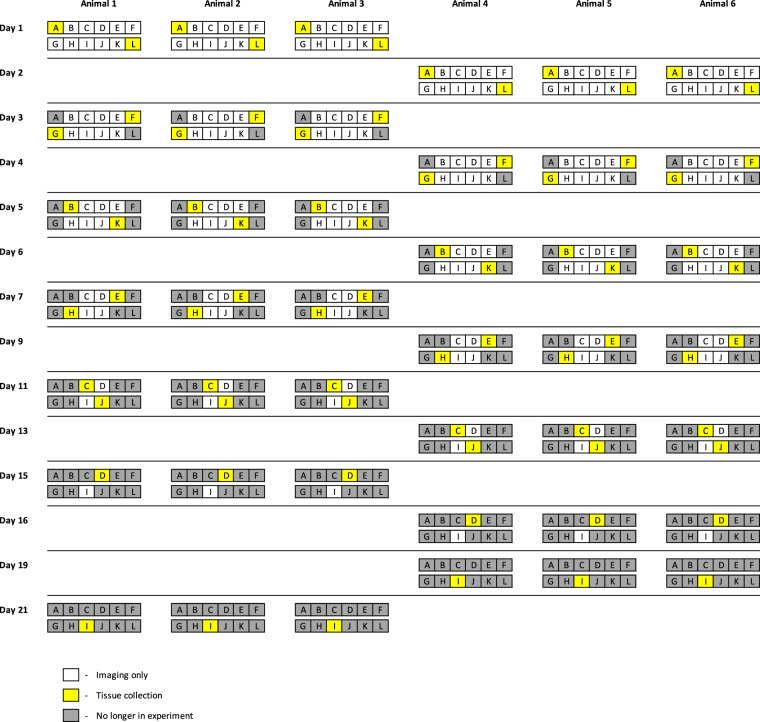
Tissue sampling outline. The wound healing experiment was conducted with 6 animals, each having 12 wounds. On each sampling day, tissue samples were collected from 3 animals, from either 1 or 2 wounds per animal. After tissue collection, the wound was removed from the experiment and no longer participated in it.

**Fig. 2:**
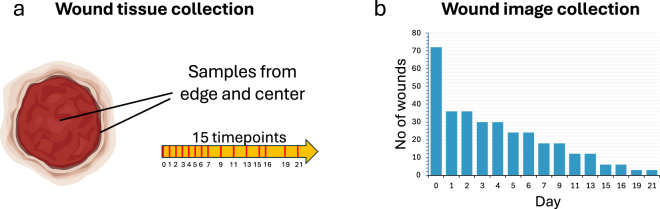
Data collection outline. (**a**) Samples from wounds were collected at 15 timepoints either from edge or center location and sent for RNAseq analysis. (**b**) Photographs were taken from intact wounds only, thus the number of photographed wounds diminished as time progressed.

Additionally, 3.5 mm biopsy samples and topical antibiotic (Animax Ointment, Dechra Pharmaceuticals, Northwich, UK) was applied to the biopsy site to prevent infection. The biopsy samples were taken from the wound edges and centers of 1 or 2 wounds at each time point in the anesthetized animals. A summary of the tissue sampling protocol is provided in Figs. 1 and 2. Tissue samples were stored in RNALater at 4 degrees until processed.

As the number of intact wounds diminished as the experiment progressed, the image collection contains more images from the earlier days and fewer images from the later days (Fig. 2b).

### RNAseq

RNAseq analysis was performed by Novogene (Beijing, China). Sample labels were anonymized, the labels were encoded to avoid bias. The mRNA was extracted from these samples with the RNeasy fibrous tissue mini kit (Qiagen) and > 200 ng mRNA/sample were sent to Novogene for RNA sequencing. The followings were done by Novogene: 1. RNA Sample QC 2. Library Preparation (Poly-A Enrichment) 3. Sequencing (Illumina – PE150 – 20 M Paired Reads) 4. Raw Data (FASTQ Files) 5. Gene counts in zipped .xls file.

Quality control (QC) metrics were evaluated by Novogene using standard procedures. Adapter-containing reads, reads with poly-N, and low-quality reads were removed from the raw FASTQ files. Quality statistics, including Q20, Q30, and GC content of the clean data, were calculated. Among all 150 samples, Q20 values ranged from 95.26% to 98.21%, Q30 from 89.02% to 95.04%, and GC content from 49.58% to 55.76%.

Reads were aligned to the reference genome by Novogene. The percentage of reads mapped to the genome ranged from 78.85% to 92.07% across samples. The unique mapping rate ranged from 76.68% to 88.66%, and the multiple mapping rate from 1.88% to 1.95%. Read duplication rates were not provided by Novogene and therefore are not reported.

Gene counts file was extracted and saved in .xlsx format.

## Data Records

The wound integrative dataset, consisting of wound images and gene expression data, is publicly available^14^ at the Dryad database.

Gene expression values and corresponding sample metadata are combined in a single processed file, “PigWoundRNAData.xlsx”. Each column represents a sample, and each row represents a gene. The first column contains gene names; the last column contains Ensembl IDs. Sample-level metadata is appended in the last four rows of the file. The encoding of sample labels is explained in the table “RNAseq Codes.xlsx”. This table contains the pig ID, wound number, day of collection from the wound onset, and wound location label (center or edge).

Files with source readings of RNA sequences are provided at NCBI Sequence Read Archive^15^. Gene expression data are also accessible through NCBI Gene Expression Omnibus (GEO) database^16^.

## Technical Validation

### Validation of gene expression data

#### Comparison of gene expression in wound edge and center samples

A total of 150 samples were collected at 15 time points. Six samples were collected on the day of wounding (day 0) and consisted of the entire excised tissue, thus did not have a wound center or edge label. On days 1–7, 9, 11, and 13, six samples were collected from the wound edge and six from the wound center. On days 15, 16, 19, and 21, three samples were collected from the edge and three from the center. Thus, the samples represent either six or three replicates of one of the 29 wound tissue states. Similarity in gene expression is expected between samples from the same day and location.

For validation, we calculated Pearson correlation coefficients between replicates. The results are presented as 29 matrices (Fig. 4), where the number at the intersection of the i-th column and the j-th row reflects the correlation between the i-th and j-th samples, making the matrices symmetrical. The closer the correlation coefficient is to 1, the more similar the gene expression of the two samples.

**Fig. 3:**
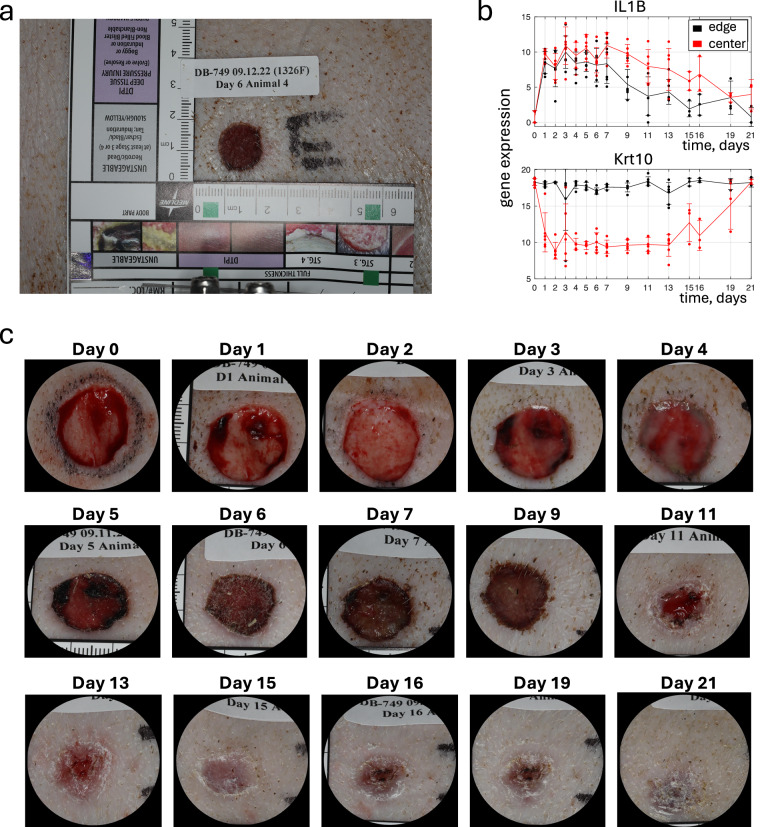
Examples of data. (**a**) Wound image including a ruler and label. (**b**) Transcriptomic time series for two genes, with gene expression plotted as *log*_2_(1 + *counts*). (**c**) Time series of cropped wound images: wounds “I” for Animals 3 and 6 (see Fig. 1).

**Fig. 4:**
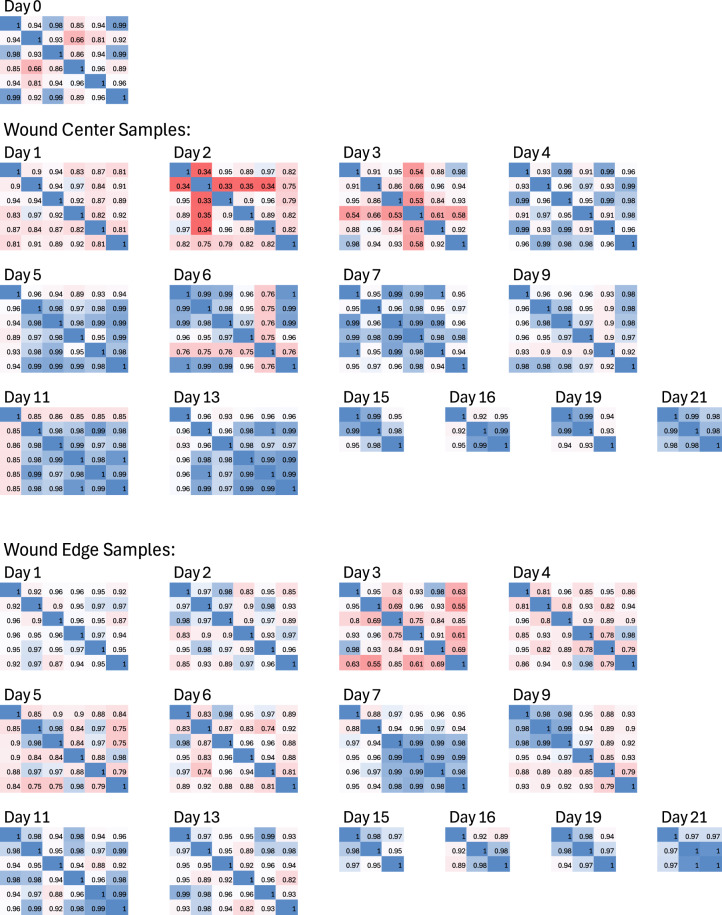
RNAseq data validation. Pearson’s correlation matrices visualize the correlation values between samples. Each matrix corresponds to samples from the same day and location (edge or center of the wound). If each sample belongs to the same cell type, the correlation should exceed 0.9. As shown here, there is some heterogeneity in the prevalent cell types, and some samples (e.g., day 2 from the wound center) are outliers. Analysis of the corresponding data should account for this heterogeneity.

It is seen from Fig. 4 that several samples deviate from other replicates, notably, the samples collected from the wound center on day 2 (sample 2) and day 3 (sample 4), as well as sample 5 from the wound edge collected on day 3. Such differences could be due to natural heterogeneity of the wound tissue or errors in sample collection. This should be considered in further data analysis.

## Usage Notes

Examples of wound image gene expression timeline and cropped wound image timeseries are presented in Fig. 3. Readers can access the expression time series plots of other genes through an online tool at https://geneexpressionsearch.pythonanywhere.com. (see github.com/mkesapra/GeneExpressionSearch for the code of the online gene plotting tool).

## Data Availability

The RNA sequencing data generated in this study have been deposited in the NCBI Sequence Read Archive (SRA) under accession code SRP591687 (https://identifiers.org/ncbi/insdc.sra:SRP591687; BioProject accession number PRJNA1276066; https://www.ncbi.nlm.nih.gov/bioproject/?term=PRJNA1276066). Gene expression data are available in the NCBI Gene Expression Omnibus (GEO) under accession code GSE305817 (https://identifiers.org/geo/GSE305817). The integrative dataset, including gene expression data and associated wound image data, is available at Dryad (10.5061/dryad.0rxwdbsbr).

## Code availability

No custom code was used to generate or process the data described in this manuscript.

## References

[1] Schilling, J. A. Wound healing. *Surg Clin North Am* **56**, 859–74, 10.1016/s0039-6109(16)40983-7 (1976). 959948 10.1016/s0039-6109(16)40983-7

[2] Singer, A. J. & Clark, R. A. Cutaneous wound healing. *N Engl J Med* **341**, 738–46, 10.1056/nejm199909023411006 (1999). 10471461 10.1056/NEJM199909023411006

[3] Kagawa, S. *et al*. The time-course analysis of gene expression during wound healing in mouse skin. *Leg Med (Tokyo)* **11**, 70–5, 10.1016/j.legalmed.2008.09.004 (2009). 18974019 10.1016/j.legalmed.2008.09.004

[4] Chen, L. *et al*. Positional differences in the wound transcriptome of skin and oral mucosa. *BMC Genomics* **11**, 471, 10.1186/1471-2164-11-471 (2010). 20704739 10.1186/1471-2164-11-471 PMC3091667

[5] Feezor, R. J. *et al*. Temporal patterns of gene expression in murine cutaneous burn wound healing. *Physiol Genomics* **16**, 341–8, 10.1152/physiolgenomics.00101.2003 (2004). 14966252 10.1152/physiolgenomics.00101.2003

[6] Sullivan, T. P., Eaglstein, W. H., Davis, S. C. & Mertz, P. The pig as a model for human wound healing. *Wound Repair Regen* **9**, 66–76 (2001). 11350644 10.1046/j.1524-475x.2001.00066.x

[7] FDA. Guidance for industry chronic cutaneous ulcer and burn wounds - developing products for treatment (2006). 10.1046/j.1524-475x.2001.00258.x 11679134

[8] Lindblad, W. J. Considerations for selecting the correct animal model for dermal wound-healing studies. *J Biomater Sci Polym Ed* **19**, 1087–96, 10.1163/156856208784909390 (2008). 18644233 10.1163/156856208784909390

[9] Ansell, D. M., Holden, K. A. & Hardman, M. J. Animal models of wound repair: Are they cutting it? *Exp Dermatol* **21**, 581–5, 10.1111/j.1600-0625.2012.01540.x (2012). 22775993 10.1111/j.1600-0625.2012.01540.x

[10] Gordillo, G. M. *et al*. Preclinical models of wound healing: Is man the model? proceedings of the wound healing society symposium. *Adv Wound Care (New Rochelle)* **2**, 1–4, 10.1089/wound.2012.0367 (2013). 24527316 10.1089/wound.2012.0367 PMC3840478

[11] Grada, A., Mervis, J. & Falanga, V. Research techniques made simple: Animal models of wound healing. *J Invest Dermatol* **138**, 2095–2105.e1, 10.1016/j.jid.2018.08.005 (2018). 30244718 10.1016/j.jid.2018.08.005

[12] Parnell, L. K. S. & Volk, S. W. The evolution of animal models in wound healing research: 1993-2017. *Adv Wound Care (New Rochelle)* **8**, 692–702, 10.1089/wound.2019.1098 (2019). 31827981 10.1089/wound.2019.1098 PMC6904936

[13] Jin, L. *et al*. A pig BodyMap transcriptome reveals diverse tissue physiologies and evolutionary dynamics of transcription. *Nat Commun* **12**, 3715, 10.1038/s41467-021-23560-8 (2021). 34140474 10.1038/s41467-021-23560-8 PMC8211698

[14] Zlobina, K. *et al*. Wound image and transcriptome datasets of swine acute wounds. *Dryad*10.5061/dryad.0rxwdbsbr (2025).

[15] *NCBI Sequence Read Archive* https://identifiers.org/ncbi/insdc.sra:SRP591687 (2025).

[16] *NCBI Gene Expression Omnibus* https://identifiers.org/geo/GSE305817 (2025).

